# A Novel Multi-Slope Chirp Modulation and Demodulation with Instantaneous Chirp Rate Estimation

**DOI:** 10.3390/s26092603

**Published:** 2026-04-23

**Authors:** Apiwat Magkeethum, Sukkharak Saechia, Paramote Wardkein

**Affiliations:** 1 School of Engineering, King Mongkut’s Institute of Technology Ladkrabang, Bangkok 10540, Thailand; 67016104@kmitl.ac.th (A.M.); paramote.wa@kmitl.ac.th (P.W.); 2 Faculty of Science and Technology, Phranakhon Si Ayutthaya Rajabhat University, Ayutthaya 13000, Thailand

**Keywords:** LoRa, LPWANs, chirp modulation and demodulation, chirp spread spectrum, non-coherent detection

## Abstract

The growth of Internet of Things (IoT) applications is driving demand for Low-Power Wide-Area Networks (LPWANs) to support higher data rates with the same energy efficiency. While Long Range (LoRa) provides excellent noise immunity and receiver sensitivity, its data rate might be insufficient for some applications, including those real-time applications in which LoRa is required to have infrequent transmissions to maintain low power consumption. In this paper, a novel modulation is introduced to address these limitations by utilizing narrowband chirp to represent a data symbol with chirp slopes, called a multi-slope chirp signal. At the receiver, a new blind non-coherent detection technique is also presented to recover the proposed signal. The simulation results confirm that the proposed scheme can successfully transmit information at 2 to 4 bits per symbol, and when compared to LoRa SF 6, it reduces the Time-on-Air (ToA) by half and also achieves an improvement in spectral efficiency in the frequency domain.

## 1. Introduction

LoRa offers wireless connectivity to most IoT devices across LPWANs and is broadly used for connecting long-distance devices which often typically require slow and infrequent communication to exchange sensor data or short parameters [1]. LoRa was originally designed to operate in regional sub-GHz ISM bands, such as 868 MHz in Europe and 915 MHz in North America. More recently, Semtech released a 2.4 GHz variant (SX1280) enabling a universally deployable chipset that functions across regions without hardware adaptation [2]. As the demand for IoT grows rapidly, the number of applications which cannot find an optimal spectral efficiency between LoRa and Wi-Fi also increases. Efficient modulation techniques are thus needed to ensure a proper data transmission rate and handle the short communication time.

In conventional digital communication standards, increasing the transmission rate is typically achieved by utilizing quadrature modulation such as Phase-Shift Keying (PSK) or Quadrature Amplitude Modulation (QAM) which require more power to overcome the noise at a high transmission rate [3]. Moreover, the bandwidth occupied by the quadrature modulation scheme is directly proportional to the symbol rate, making the bandwidth easily to be fully utilized [4]. The bandwidth constraints also apply to LoRa communication. Since LoRa relies on chirp spread spectrum (CSS) to achieve extraordinary noise immunity and ultra-low power consumption, it must spread the information across a wide frequency band and time domain simultaneously [5]. In contrast, the downsides of this approach are a very low data rate and the fact that the Time-on-Air (ToA) of LoRa is incredibly long to finish the transmission [6]. Meanwhile, it also occupies the bandwidth during that time. Thus, the multiplexing of LoRa is difficult to achieve and creates challenges for the large-scale communication of many devices in a LoRa network [7].

The main contribution of this study is a new modulation scheme with a transmitter and receiver block diagram utilizing chirp signals in a new physical layer approach called the multi-slope chirp signal to achieve the benefits of higher spectral efficiency and faster Time-on-Air than LoRa. It is similar to LoRa but not the same. While LoRa uses cyclic-shifted chirp symbols where the data is encoded by varying the starting frequency [8], the proposed scheme encodes information by the different slopes of the chirp symbols, with several techniques added, and the bandwidth and center frequency of the chirp signal can remain consistent regardless of any payload. Moreover, the proposed scheme can adjust the noise immunity based on the occupied bandwidth. Demodulation schemes are also proposed in order to recover the multi-slope chirp signal. It is explained in two different channels. In a lossless channel, the DeChirp algorithm is used for recovery, while in an AWGN channel, SAGC-2 is used instead. All of the proposed schemes in this study have mathematical models to prove the concept alongside the simulation that leads to the proof; moreover, the proposed multi-slope chirp symbol can be constructed on a commercialized STM32 microcontroller.

This paper is organized as follows. In Section 1, the background and limitations of LoRa are addressed and discussed. Section 2 provides a review of the principles of linear chirp signal and multi-slope chirp signal and its bandwidth. In Section 3, the proposed multi-slope chirp symbol encoding and decoding techniques are presented. Section 4 illustrates the simulations and results of the proposed systems. Finally, a discussion is provided in Section 5.

## 2. Principles

### 2.1. Single-Slope Chirp Signal

**Figure 1:**
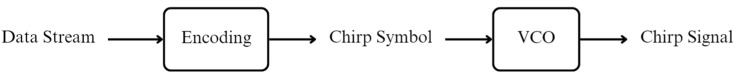
Chirp signal modulation block diagram.

Chirp modulation is an analog modulation technique because of its continuous frequency sweeping in a linear fashion [9]. This sweeping characteristic provides robustness against narrowband noise, multipath interference, and Doppler effects [10], which makes it useful for radar and sonar systems. Additionally, single-slope chirp modulation can be adapted and integrated into digital communication systems such as LoRa [11] and digital passive radar systems. To create a chirp signal, the technique is quite simple: a data stream is encoded into a chirp symbol, and then the symbol is taken to the voltage-controlled oscillator (VCO) to vary the output voltages according to the input information, as depicted in Figure 1. In practice, a frequency modulation (FM) modulator is used since it has VCO built in, and then the chirp signal is present at the output of the modulator.

In modern transceivers, chirp signal generation is often implemented by direct digital synthesis (DDS) or digitally controlled oscillators or in a software-defined radio (SDR) unit, reducing usage of analog voltage-controlled oscillators [12].

**Figure 2:**
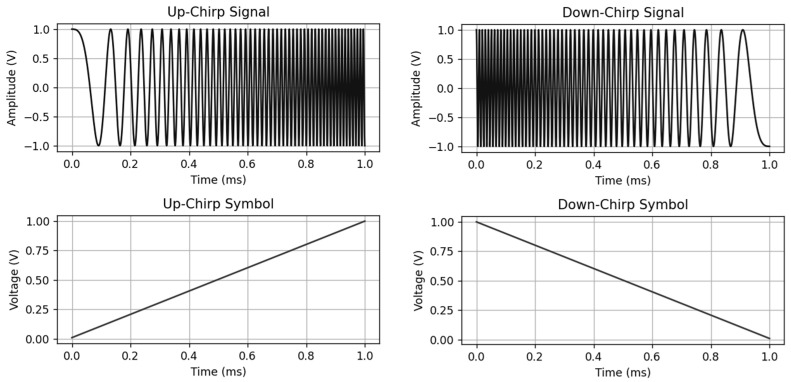
Single-slope chirp signal (**top**) and single-slope chirp symbol (**bottom**).

A single-slope chirp symbol is a single unit encoded from binary digital data, either ‘1’ or ‘0’. They are represented by an increment or decrement in voltage in a linear manner denoted by $m\left(t\right)$, with $k_{c}$ as its slope to define the upward or downward behavior of the symbol [13], as shown in Equation (1).

(1) $$m\left(t\right)=v_{0}+k_{c}t$$

where $v_{0}$ is the initial voltage of the chirp symbol. In order to create the single-slope chirp signal, an FM modulator dynamically outputs the instantaneous frequency of the signal according to the input voltages of the single-slope chirp symbols, as shown in Figure 2.

The signal at the output of the FM modulator has a generic equation [14], shown as (2):

(2) $$s_{FM}\left(t\right)=A\cos\left(\omega_{r}t+2\pi k_{f}\int_{0}^{t}m\left(\tau \right)d\tau \right)$$

where $s_{FM}\left(t\right)$ is the signal at the output of the FM modulator, A is the amplitude, $\omega_{r}$ is the free-running angular frequency (rad/s) of the VCO used in FM modulator, and $k_{f}$ is the VCO gain (Hz/V). When $m\left(t\right)$ is taken to the FM modulator, (3) is obtained at the output.

(3) $$s_{FM}\left(t\right)=A\cos\left(\left(\omega_{r}+2\pi k_{f}v_{0}\right)t+\pi k_{f}k_{c}t^{2}\right)$$

A chirp symbol with slope $k_{c}$ and period of $T_{s}$ seconds is used to determine the bandwidth of the chirp signal, so the bandwidth results in (4).

(4) $$BW=f\left(T_{s}\right)-f\left(0\right)=\left|k_{f}k_{c}T_{s}\right|$$

When the time period is a constant, the slope and the time period will be canceled out. By substituting $k_{c}$ as in (5), the bandwidth can be rewritten as (6). Under this condition, the bandwidth of the chirp signal depends on the amplitude of the chirp symbol instead of the symbol period [15].

(5) $$k_{c}=\frac{v_{p}-v_{0}}{T_{s}}$$

(6) $$BW=\left|k_{f}V_{pp}\right|$$

where $V_{pp}$ is the peak-to-peak amplitude of the chirp symbol. From Equation (6), the bandwidth is no longer related to the symbol period but to the amplitude of the symbol and VCO gain instead. By this property, it can be used to develop the multi-slope chirp signal in order to achieve higher spectral efficiency.

### 2.2. Multi-Slope Chirp Signal

A multi-slope chirp symbol was first introduced in [16] and was called a variable-slope chirp spread spectrum (VS-CSS). The authors discussed the error performance of the signal on the optimum receiver but did not provide the transmitter architecture. Therefore, in this article, a block diagram of the transmitter, receiver, and bandwidth control techniques is proposed. The core idea of a multi-slope chirp symbol is to represent n-bit of data to $2^{n}$ different values of $k_{c}$. A single unit of the symbol is a linear equation as in (7), with a slope of $k_{c}$ corresponding to the encoding bits; the higher the symbol value, the higher the slope of $k_{c}$.

**Figure 3:**
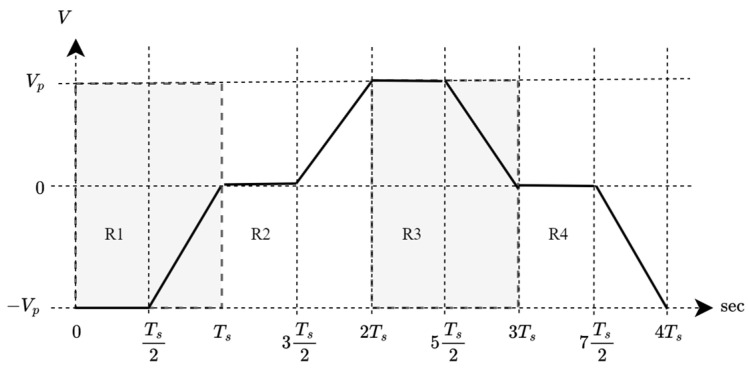
A complete cycle of 2-bit multi-slope chirp symbols.

Since the initial voltage of chirp symbols affects the center frequency as in (3), random payload will make the frequency random, causing the center frequency and bandwidth to be inconsistent. Therefore, a redundant bit is added after every data bit only to control the voltage. A stable multi-slope chirp symbol needs to be constructed in a cycle, as shown in Figure 3, where the initial and ending voltages are identical.

To do so, four regions need to be satisfied, running from R1 to R4, and each region determines the voltage directions as follows: R1 initiates the amplitude at $-V_{p}$ and continuously raises the amplitude up to 0 V, R2 further increases the amplitude up to $V_{p}$, R3 reduces the amplitude back to 0V, and finally, R4 drops the amplitude back to the initial value.

**Figure 4:**
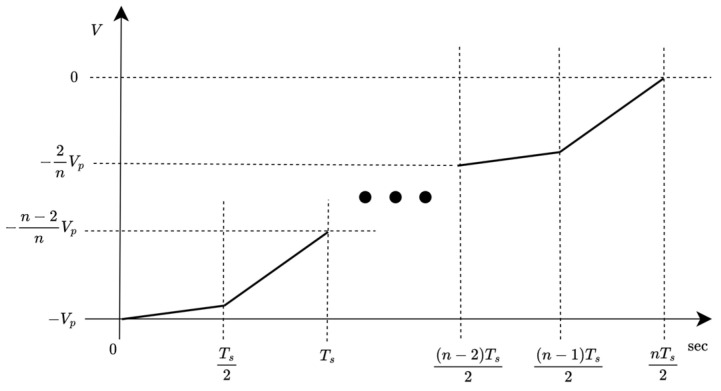
R1 region of n-bit multi-slope chirp symbols.

A higher value of the n-bit multi-slope chirp symbol can be constructed to increase spectral efficiency; therefore, the R1 region for the n-bit symbol is provided as an instruction in Figure 4. The n-bit multi-slope chirp symbol also needs to be covered through the whole region to complete a cycle; otherwise, it will become unstable.

An equation for a multi-slope chirp symbol can be expressed as (7), where i represents the $i^{th}$ symbol in the cycle. Each symbol in the cycle has $k_{ci}$ as its slope and $v_{i}$ as its y-interception point. Since one information symbol needs one redundant symbol to control the voltages, the period of one chirp symbol is reduced by half of the information symbol. Thus, a single chirp symbol corresponds to a single value of $k_{ci}$.

(7) $$m_{i}\left(t\right)=k_{ci}t+v_{i};\;\frac{\left(i-1\right)T_{s}}{2}\leq t\leq \frac{iT_{s}}{2}\;$$

When (7) is taken to the FM modulator, a multi-slope chirp signal at the $i^{th}$ symbol or $s_{i}\left(t\right)$ is obtained at the output of the modulator and can be expressed as (8).

(8) $$s_{i}\left(t\right)=A\cos\left(\omega_{r}t+2\pi k_{f}\int_{0}^{t}m_{i}\left(\tau \right)d\tau \right)$$

The integration in (8) can be rewritten in a more specific way according to Figure 3, focusing on where the $i^{th}$ symbol is being integrated, as in (9).

(9) $$\int_{0}^{t}m_{i}\left(\tau \right)d\tau =\phi_{i-1}+\int_{\frac{\left(i-1\right)T_{s}}{2}}^{t}m_{i}\left(\tau \right)d\tau \;$$

where $\phi_{i-1}$ is the known integrated value of the previous symbol and can be expressed as (10), so it is clearly seen as a constant. For example, in Figure 3, if R2 is being integrated, it must start from the end of R1, and the end of R1 is $\phi_{i-1}$.

(10) $$\phi_{i-1}=\sum_{j=1}^{i-1}\left[\int_{\frac{\left(j-1\right)T_{s}}{2}}^{\frac{jT_{s}}{2}}m_{j}\left(t\right)dt\right]$$

By substituting (7) with (9), a result of integrated $m_{i}\left(t\right)$ is obtained as (11).

(11) $$\int_{0}^{t}m_{i}\left(\tau \right)d\tau =C_{i}+v_{i}t+\frac{k_{ci}t^{2}}{2}$$

where $C_{i}$ is the summation of all constant terms after the integration, which can be expressed as (12).

(12) $$C_{i}=\phi_{i-1}-\frac{k_{ci}{\left(i-1\right)}^{2}T_{s}^{2}}{8}-\frac{v_{i}\left(i-1\right)T_{s}}{2}$$

Finally, by having (11), the equation of the multi-slope chirp signal can be obtained and be expressed as (13).

(13) $$s_{i}\left(t\right)=A\cos\left(\omega_{i}t+\pi k_{f}k_{ci}t^{2}+\theta_{i}\right)$$

$\omega_{i}$ and $\theta_{i}$ are a new initial frequency and the phase of each of the signals. $\theta_{i}$ is a phase memory which makes the signal have a continuous phase property, and $\omega_{i}$ makes the signal have a continuous frequency. The two equations are expressed as (14) and (15), respectively.

(14) $$\omega_{i}=\omega_{r}+2\pi k_{f}v_{i}$$

(15) $$\theta_{i}=2\pi k_{f}C_{i}$$

The instantaneous frequency of a multi-slope chirp signal of $i^{th}$ symbol $f_{i}\left(t\right)$ is found in (16) by taking the derivative to the phase of (13).

(16) $$f_{i}\left(t\right)=\frac{\omega_{r}}{2\pi }+k_{f}m_{i}\left(t\right)$$

By locating the maximum and minimum voltages, the bandwidth of the multi-slope chirp signal can be derived and with the constraints that the average voltage of the symbol is zero, then, $V_{pp}=2V_{p}$ [4].

(17) $$BW=\left|k_{f}V_{pp}\right|$$

From (17), it is verified that the bandwidth of the multi-slope chirp signal is independent from the symbol period but is related to the amplitude of the symbol and the VCO gain instead. Its bandwidth is equal to the single-slope chirp bandwidth in (6). In conclusion, the multi-slope chirp signal can be used to increase the data rate while retaining the bandwidth.

**Figure 5:**
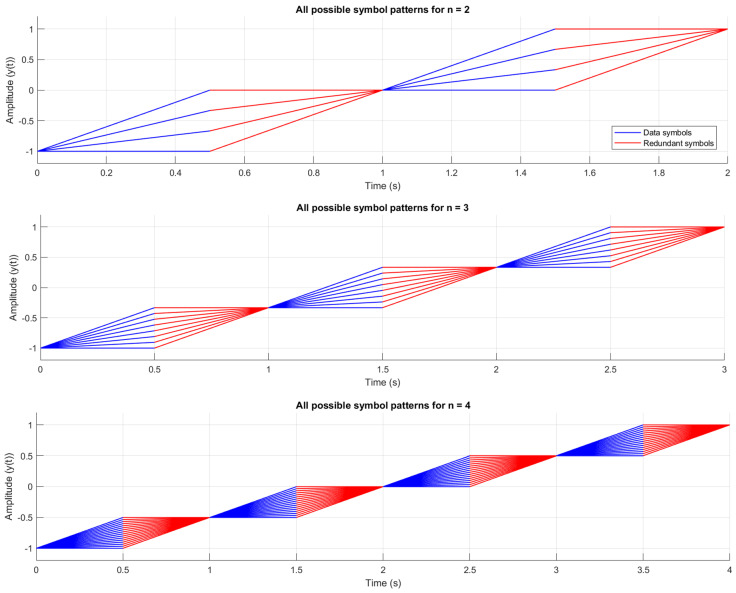
All possible patterns of n-bit multi-slope chirp symbols in the first half cycle.

The equation of −1 V to 1 V n-bit multi-slope chirp symbol in the first half cycle and its redundant symbols, along with the slope $k_{ci,n}$ and the y-intercept point $v_{i,n}$, can be found in Table 1, where $d_{i}$ denotes the symbol value, ranging from 0 to $2^{n}-1$, and j is the segment, ranging from 1 to n; one segment contains two symbols as a pair in blue and red trace, as shown in Figure 5. All possible symbol patterns are visualized in Figure 5.

## 3. Methods

In this section, the generation of the multi-slope chirp symbol and signal, including encoding and modulation, as well as their reception, including demodulation and decoding in both lossless and AWGN channels, are discussed. Block diagrams and technical descriptions are provided in this section.

### 3.1. Multi-Slope Chirp Symbol Encoding and Modulation

**Figure 6:**
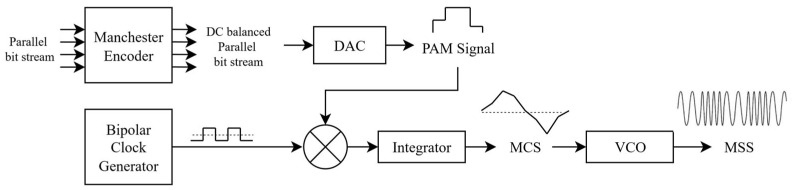
Multi-slope chirp signal generation block diagram.

The generation of the multi-slope chirp symbol requires six components, as shown in Figure 6: a Manchester encoder, a digital-to-analog converter (DAC), a bipolar clock generator, a multiplier, an integrator, and a VCO.

Firstly, n parallel binary streams with a bit period of $T_{s}$ are Manchester-encoded to create redundant bits in order to adjust the voltages to the constant, as explained in Section 2, to achieve center frequency balance and bandwidth control. Next, the parallel Manchester-encoded stream is converted to a single PAM signal by DAC and then multiplied with the bipolar clock, a clock signal that has amplitude ±1; the resulting signal at the output of the multiplier is called Alternating PAM (APAM). At this stage, the PAM signal is periodically inverted corresponding to the period of the bipolar clock. The period of the bipolar clock is defined as $2nT_{s}$ to match the region specification.

Next, APAM is integrated and its positive side keeps increasing until it reaches the maximum amplitude of $V_{p}$; at this point, the integration begins to decrease because the integrand is now negative due to APAM being inverted at point $nT_{s}$ in Figure 3. It keeps decreasing until $-V_{p}$ at point $2nT_{s}$ completing the cycle. When a full cycle is complete, a multi-slope chirp symbol is successfully constructed. Finally, it is modulated to a multi-slope chirp signal by VCO and is ready to be transmitted out.

The bipolar clock is the part that is used to determine the n-bit of the multi-slope chirp signal; as mentioned, the bipolar clock period can be increased or decreased to collect more or fewer symbols. Since the maximum and minimum amplitudes are fixed, the slopes are more tightened, and it is harder to detect if the same period of the clock is used, so the clock must be adjusted according to the n-bit. However, the more symbols it accumulates, the more difficult it is for the receiver to detect the slope differences, leading to a higher error rate and vice versa. This technique can be related to the modulation order concept in quadrature modulation.

### 3.2. Multi-Slope Chirp Symbol Demodulation and Decoding

**Figure 7:**
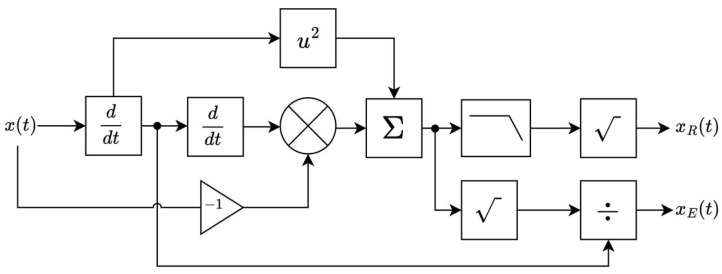
A modified sinusoidal automatic gain control (SAGC-2) block diagram.

The main component involved in both the demodulation and decoding processes is called modified sinusoidal automatic gain control (SAGC-2). SAGC was first introduced in [17] and can equalize and demodulate AM, FM [18], and chirp signal [19]. SAGC-2 has been modified specifically to process the multi-slope chirp signal, as shown in Figure 7.

There are 2 outputs from SAGC-2: the equalized output denotes as $x_{E}\left(t\right)$ and the demodulated output as $x_{R}\left(t\right)$. SAGC-2 will be implemented in both scenarios when the channel is lossless and lossy. For the lossless channel, an algorithm called DeChirp will be used, which is newly introduced in this paper; it combines chains of mathematical operations with SAGC-2 equalizer to extract the information from the multi-slope chirp signal directly. DeChirp takes the multi-slope chirp signal as the input and retrieves the information in APAM format.

**Figure 8:**
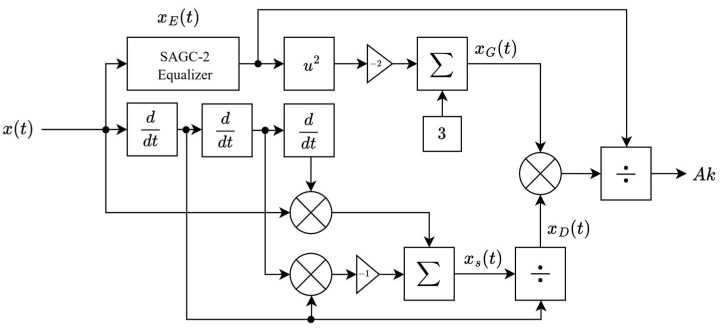
DeChirp block diagram.

Another advantage of DeChirp is that it can be applied to any chirp signal without having prior knowledge of the chirp symbol or chirp signal characteristics or even the symbol rate of the information (blind property). The downside of DeChirp, however, is that it is still very hypothetical and can only be used in a lossless channel. The block diagram of DeChirp is shown in Figure 8, and its operation is expressed in Equations (18)–(27).

To prove the DeChirp algorithm, a multi-slope chirp signal shown in (15) is initiated again in (18) with a different format for easier derivation by giving $\phi \left(t\right)=\omega_{i}t+\frac{kt^{2}}{2}+\theta_{i}$.

(18) $$x\left(t\right)=A\cos\left(\phi \left(t\right)\right)$$

where A is the amplitude and $k=2\pi k_{f}k_{ci}$. By taking the first, second, and third derivative of (18), $x\left(t\right)x^{\prime \prime \prime }\left(t\right)$ and $x^{\prime }\left(t\right)x^{\prime \prime }\left(t\right)$ can be calculated and the results are in (19) and (20), respectively.

(19) $$x\left(t\right)x^{\prime \prime \prime }\left(t\right)=\frac{A^{2}{\phi^{\prime }\left(t\right)}^{3}\sin\left(2\phi \left(t\right)\right)}{2}-3A^{2}k\phi^{\prime }\left(t\right)\cos^{2}\left(\phi \left(t\right)\right)$$

(20) $$x^{\prime }\left(t\right)x^{\prime \prime }\left(t\right)=\frac{A^{2}{\phi^{\prime }\left(t\right)}^{3}\sin\left(2\phi \left(t\right)\right)}{2}+A^{2}k\phi^{\prime }\left(t\right)\sin^{2}\left(\phi \left(t\right)\right)$$

Performing (19) minus (20) results in $x_{s}\left(t\right)$ expressed in (21).

(21) $$x_{s}\left(t\right)=-A^{2}k\phi^{\prime }\left(t\right)\left(2+\cos\left(2\phi \left(t\right)\right)\right)$$

Taking $x^{\prime }\left(t\right)$ divided by $x_{s}\left(t\right)$ in (21) results in $x_{D}\left(t\right)$, expressed in (22).

(22) $$x_{D}\left(t\right)=\frac{\sin\left(\phi \left(t\right)\right)}{Ak\left(2+\cos\left(2\phi \left(t\right)\right)\right)}$$

Equation (22) does not introduce any discontinuity points; therefore, the division is safe to perform. Next, $x_{E}\left(t\right)$ is taken from SAGC-2, with the equation shown in (23), and given the fact that ${\phi \prime \left(t\right)}^{2}\gg k$, the fraction term can be neglected, which results in the constant amplitude chirp signal in (24).

(23) $$x_{E}\left(t\right)=\frac{x^{\prime }\left(t\right)}{\sqrt{x^{\prime }{\left(t\right)}^{2}-x\left(t\right)x^{\prime \prime }\left(t\right)}}=\frac{-\sin\left(\phi \left(t\right)\right)}{\sqrt{1+\frac{k\sin\left(2\phi \left(t\right)\right)}{2\phi^{\prime }{\left(t\right)}^{2}}}}$$

(24) $$x_{E}\left(t\right)\approx -\sin\left(\phi \left(t\right)\right)$$

Squaring (24), then multiplying it by −2, and then adding 3 results in $x_{G}\left(t\right)$ expressed in (25).

(25) $$x_{G}\left(t\right)\approx 2+\cos\left(2\phi \left(t\right)\right)$$

From (25), the obtained $x_{G}\left(t\right)$ is exactly the same as the denominator of (22); by substituting it, as well as $x_{E}\left(t\right)$, to the numerator results in (26).

(26) $$Ak\approx \frac{-x_{E}\left(t\right)}{x_{G}\left(t\right)x_{D}\left(t\right)}$$

Finally, every newly created variable in (26) is substituted back to the original $x\left(t\right)$, so the complete equation of the DeChirp algorithm is expressed in (27), and by applying the Teager–Kaiser energy operator $\Psi \left(\cdot \right)$, the equation can be reduced to (28) [20].

(27) $$Ak\approx \left[x^{\prime }\left(t\right)x^{\prime \prime }\left(t\right)-x\left(t\right)x^{\prime \prime \prime }\left(t\right)\right]\frac{\sqrt{x^{\prime }{\left(t\right)}^{2}-x\left(t\right)x^{\prime \prime }\left(t\right)}}{x^{\prime }{\left(t\right)}^{2}-3x\left(t\right)x^{\prime \prime }\left(t\right)}$$

(28) $$Ak\approx \frac{\Psi^{\prime }\left(x\left(t\right)\right)\sqrt{\Psi \left(x\left(t\right)\right)}}{\Psi \left(x\left(t\right)\right)-2x\left(t\right)x^{\prime \prime }\left(t\right)}$$

**Figure 9:**
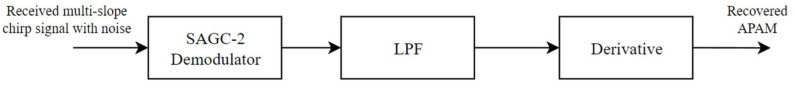
Demodulating and decoding multi-slope chirp signal over AWGN channel.

From (27), the chirp rate k, which is the correspondence of the information data, is successfully extracted. Hence, by knowing k, the information data can also be obtained. For the AWGN channel, DeChirp is not recommended to be used because the noise in the signal will be dominant after multiple differentiation processes, and without any filtering, the signal will become too noisy to detect. The appropriate way to demodulate the signal is by using a filter to clean the noise, then using SAGC-2 to demodulate the signal back to the symbol, and then decoding the information out, as shown in the block diagram in Figure 9. The demodulation process is explained in Equations (29)–(32).

The demodulation process begins at the input of the first derivative block of SAGC-2, with the equation shown in (29).

(29) $$s_{i}\left(t\right)=A\cos\left(\phi_{i}\left(t\right)+\theta_{n}\left(t\right)\right)$$

where $\phi_{i}\left(t\right)=\omega_{i}t+\frac{kt^{2}}{2}+\theta_{i}$ and $\theta_{i}\left(t\right)$ is the phase noise received. Next, the SAGC-2 processes the signal until the output of the summing block of SAGC-2 or $x_{c}\left(t\right)$, where the sum is shown in (30). SAGC-2 differentiates the signal twice but in Equation (31), the noise in the signal term is only ${\dot{\theta }}_{n}\left(t\right)$.

(30) $$x_{c}\left(t\right)=A^{2}{\left(\omega_{i}+kt+{\dot{\theta }}_{n}\left(t\right)\right)}^{2}+\frac{A^{2}\left(k+{\ddot{\theta }}_{n}\left(t\right)\right)}{2}\sin\left(2\phi_{i}\left(t\right)+\theta_{n}\left(t\right)\right)$$

Next, a Bessel low-pass filter is selected due to its maximally flat group delay property [21] to filter out the high-frequency term, and by taking the square root to the result, $x_{R}\left(t\right)$ is obtained in (31).

(31) $$x_{R}\left(t\right)=\left|A\left(\omega_{i}+k\left(t-t_{g}\right)+{\dot{\theta }}_{n}\left(t-t_{g}\right)\right)\right|$$

where $t_{g}$ is the group delay of the Bessel filter. After (29) is obtained, by taking the derivative to $x_{R}\left(t\right)$, (32) is obtained and k is finally extracted. From this step, the noise is amplified again due to the derivative, resulting in the ${\ddot{\theta }}_{n}\left(t\right)$ term.

(32) $$x_{R}^{\prime }\left(t\right)=\left|2\pi Ak_{f}k_{ci}+A{\ddot{\theta }}_{n}\left(t-t_{g}\right)\right|$$

This step marks the end of the decoding process where the slopes of the multi-slope chirp symbol are extracted. The noise term ${\ddot{\theta }}_{n}\left(t-t_{g}\right)$ is modeled as a Wide-Sense Stationary (WSS) process over the AWGN channel, and its variance is time-invariant. That is, the variance depends only on the time differences, not absolute time. Consequently, the constant group delay $t_{g}$ introduces only a time shift and does not affect the noise variance, giving $Var\left[{\ddot{\theta }}_{n}\left(t-t_{g}\right)\right]=Var\left[{\ddot{\theta }}_{n}\left(t\right)\right]$ in (33).

(33) $$\sigma_{out}^{2}=A^{2}Var\left[{\ddot{\theta }}_{n}\left(t\right)\right]$$

Given $N_{0}$ as the noise power, the PSD of the noise obtained in (33) is shown in (34).

(34) $$S_{\ddot{\theta }}\left(f\right)=\frac{{\left(2\pi f\right)}^{4}N_{0}}{2}$$

The variance of ${\ddot{\theta }}_{n}\left(t\right)$ is the integration of (34) over the frequency domain within the signal bandwidth interval in (35), and the result of noise variance is obtained in (36), where BW is the occupied bandwidth of the n-bit multi-slope chirp signal.

(35) $$Var\left[{\ddot{\theta }}_{n}\left(t\right)\right]=\int_{-\frac{BW}{2}}^{\frac{BW}{2}}\frac{{\left(2\pi f\right)}^{4}N_{0}}{2}df$$

(36) $$\sigma_{out}^{2}=\frac{\pi^{4}A^{2}BW^{5}}{10}N_{0}$$

The decision SNR is conventionally denoted as (37).

(37) $$\gamma_{D}={\left(\frac{d_{min}}{\sigma_{out}}\right)}^{2}$$

where $d_{min}$ is the minimum Euclidean distance between symbols. For the n-bit multi-slope chirp signal, it is obtained from the distance between adjacent symbols derived from Table 1, so it is substituted into (38).

(38) $$d_{min}=2\pi Ak_{f}\left(k_{c\left(i+1\right)}-k_{ci}\right)=2\pi Ak_{f}\left(\frac{4}{n\left(2^{n}-1\right)T_{s}}\right)$$

By substituting (37) and (36) to (37), the decision SNR of the n-bit multi-slope chirp signal can be obtained in (39).

(39) $$\gamma_{D}=\frac{640k_{f}^{2}}{n^{2}{\left(2^{n}-1\right)}^{2}T_{s}^{2}\pi^{2}BW^{5}N_{0}}$$

Finally, the error probability for n-bit multi-slope chirp signal $P_{e,n}$ of the proposed receiver can be found in (40), where $E_{bavg,2}=0.38T_{s}$, $E_{bavg,3}=0.35T_{s}$, and $E_{bavg,4}=0.34T_{s}$, derived from (42), which should be normalized to $\gamma_{D}$ to facilitate direct comparison under equal average energy conditions.

(40) $$P_{e,n}=Q\left(\sqrt{\frac{\gamma_{D}}{E_{bavg,n}}\times \frac{E_{bavg,n}}{N_{0}}\;}\right)$$

### 3.3. Error Performance Analysis for Optimum Receiver over AWGN Channel

A matched filter has been proven to be the optimum receiver for AWGN to maximize the SNR of the system, and in order to find the error performance of the proposed multi-slope chirp signal. The square Euclidean distance at the corresponding n-bit multi-slope chirp signal ($D_{n}^{2})$ is found by (41). $m_{j,n}\left(t\right)=m_{i,n}\left(t\right)$ but $d_{i}$ is increased by 1. So, $d_{i}$ of $m_{j,n}\left(t\right)$ equals $d_{i}+1$ of $m_{i,n}\left(t\right)$.

(41) $$D_{n}^{2}=\frac{1}{E_{bavg,n}}\int_{\left(i-1\right)T_{s}}^{\left(2n+i-1\right)T_{s}}{\left(m_{i,n}\left(t\right)-m_{j,n}\left(t\right)\right)}^{2}dt$$

where $E_{bavg,n}$ denotes the average energy of all possible symbols within the cycle of the corresponding n-bit multi-slope chirp signal; the cycle has a period of $2nT_{s}$ from Figure 5. Since the full cycle has 2n segments and all segments have $2^{n}$ possible symbols, the total symbols in the cycle are $2n2^{n}$, as shown in (42).

(42) $$E_{bavg,n}=\frac{1}{2n2^{n}}\left(\sum_{i=1}^{n}\sum_{d_{i}=0}^{2^{n}-1}\int_{\left(i-1\right)T_{s}}^{\left(2n+i-1\right)T_{s}}{\left|m_{i,n}\left(t\right)\right|}^{2}dt\right)$$

where the first inner summation is the accumulation of all possible symbols in the segments and the outer summation is the accumulation of all segments in the cycle. After (41) and (42) are found, the minimum square Euclidean distance is calculated and that value is taken to calculate the error performance of the n-bit multi-slope chirp signal as expressed in (43).

(43) $$P_{e,n,optimum}=Q\left(\sqrt{\frac{\min\left(D_{n}^{2}\right)E_{bavg}}{2N_{0}}}\right)$$

### 3.4. Self-Error Correction

The proposed modulation scheme provides symbol error correction by the redundant bits added from Manchester encoding, so the information will now be in a binary pair. The actual information for the $k^{th}$ pair is valued as $b_{i,k}$ and the redundant bit is valued as $\bar{b_{i,k}}$. When an n-bit PAM symbol is constructed, that symbol will have a value of a and b, respectively, according to $b_{i,k}$ and $\bar{b_{i,k}}$, as shown in (44) and (45), referring to odd and even symbols, respectively.

(44) $$a_{2k-1}=\sum_{i=0}^{n-1}2^{i}b_{i,k}$$

(45) $$b_{2k}=\sum_{i=0}^{n-1}2^{i}\bar{b_{i,k}}$$

Because $\bar{b_{i}}$ is the complementary of $b_{i}$, by adding (44) and (45), the result is always a constant, as shown in (46).

(46) $$a_{2k-1}+b_{2k}=2^{n}-1$$

From (46), error corrections can be performed by comparing the error distances of the two symbols in each pair. The symbol with the smallest error distance is decided by the likelihood decision rule, while the other, which that has a less reliable symbol, can be calculated instead of making the second decision on a low-confidence symbol.

Self-error correction gain can be evaluated. In order to get an error pair, both symbols must be wrong before the correction because if at least one symbol is correct, its pair symbol will automatically be corrected by the SEC. So, the only way a pair fails correction is when both symbols are simultaneously in error. If the probability of getting one wrong symbol is $P_{e}$, the probability that both independent symbols in the pair are wrong is $P_{e}^{2}$, since each symbol experiences independent noise. This logic is taken and defined in (47).

(47) $$P_{e\left(SEC\right),n}=P_{e,n}^{2}$$

From (48), the error correction gain obtained is around 4.42 dB at SER of $10^{-3}$.

## 4. Results

**Figure 10:**
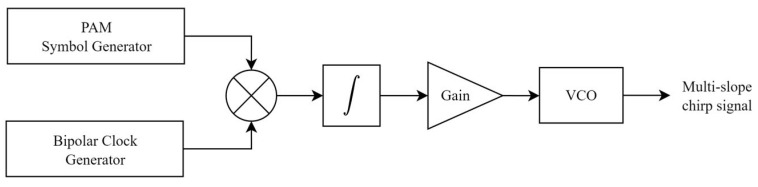
Multi-slope chirp signal transmitter.

**Figure 11:**
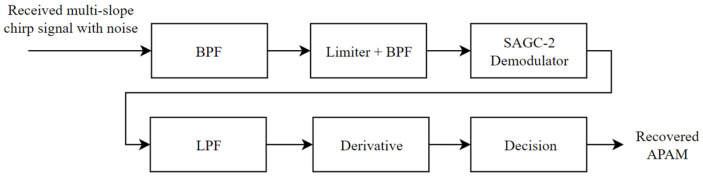
Multi-slope chirp signal receiver.

MATLAB Version 2023b is used to simulate the proposed system, including two main components: a transmitter and a receiver. An AWGN channel is used as the channel in this simulation. Figure 10 and Figure 11 illustrate the transmitter and the receiver, respectively.

### 4.1. Transmitter Encoding Simulations

The proposed transmitter is depicted in Figure 9, consisting of six components: a PAM symbol generator, a bipolar clock generator, a multiplier, an integrator, an amplifier, and a VCO.

**Figure 12:**
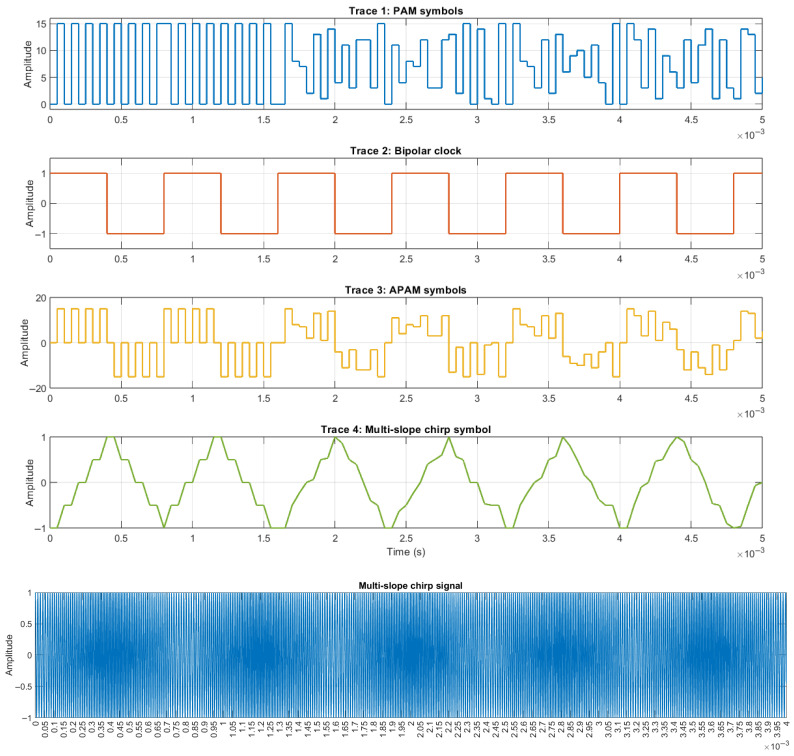
Multi-slope chirp signal generation.

The process begins inside the PAM symbol generator block consisting of two components, a Manchester encoder and a DAC. First, four parallel binary streams are generated, encoded, and then are fed to the DAC to obtain the PAM symbols at the output; the result here is shown in the first trace of Figure 12. Simultaneously, the bipolar clock signal is generated to be the inverting signal for the generation of APAM, as shown in the second trace, at a rate 16 times slower than the symbol rate of PAM in order to collect more symbols inside one multi-slope chirp symbol. Next, the PAM symbols and the clock are multiplied and result in APAM, as shown in the third trace, and then, finally, APAM is fed to the integrator to become multi-slope chirp symbols at the output, as shown in the fourth trace.

After the multi-slope chirp symbols have been generated, the amplitude of the symbols may vary due to the different symbol periods on integration; level adjustment should be performed within 2 Vpp to control the bandwidth. Finally, the VCO will be used to generate the multi-slope chirp signal, as shown in the last trace of Figure 12.

### 4.2. DeChirp-Based Decoding Simulations

**Figure 13:**
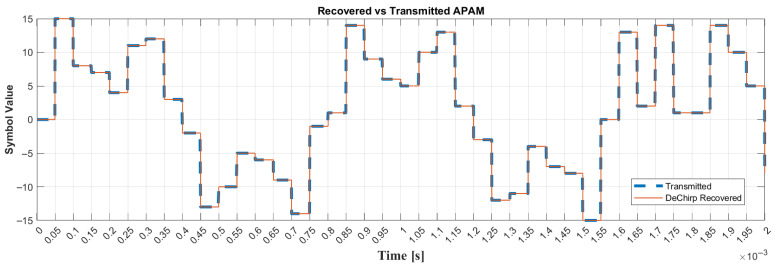
DeChirp-based multi-slope chirp signal detection.

The DeChirp algorithm is implemented as a function in MATLAB using only (27), and to prove that it works, a multi-slope chirp signal is constructed using Algorithm 1, with the lower adjustment VCO gain set to 7 kHz/V and a symbol rate of 20 kHz from a Manchester-encoded chirp symbol amplitude of 2 Vpp, with no offset. When successfully constructed, it is decoded with the DeChirp function immediately using Algorithm 2, and the result is depicted in Figure 13, where the solid line is APAM recovered by DeChirp and the dashed line is the transmitted APAM.
**Algorithm 1.** Multi-Slope Chirp Signal Generation**Inputs:** symbol_rate, bit_per_symbol **Output:** n-bit multi-slope chirp signalDefine the symbol_rate as ${1\times 10}^{4}$ and define bit_per_symbol as n ranging from 2 to 4Compute the number of generated bits N from the symbol rate and the time vector t, yielding N=500Generate random binary matrix **B** size of n×NApply Manchester encoding to **B**, producing the matrix **M** of size n×2N, right now the symbol_rate is increased twiceConvert each column of **M** from binary to decimal, resulting in a row vector **PAM** of length 2NMultiply each element of **PAM** by ${\left(-1\right)}^{k}$, starting from k=0 and adding k by one on every 2n elements, yielding a row vector **APAM** length 2NMap the **APAM** to t, resulting in the time-indexed row vector **APAM_t**Generate the multi-slope chirp symbol by integrating APAM_t with respect to tNormalize the resulting chirp symbol to a peak-to-peak amplitude of 2 V.Create a VCO with free-running frequency of 1 MHz and have VCO gain of 20 kHz/VMap the chirp symbol to the VCO, resulting in the instantaneous frequency of the VCO ($f_{VCO}$)Calculate the instantaneous phase of the VCO by integrating $f_{VCO}$ with respect to tGenerate the multi-slope chirp signal by applying the cosine operator to the instantaneous phase

As the result shown in Figure 13, DeChirp successfully extracts the information from the multi-slope chirp signal even though the bandwidth of the signal is 17.395 kHz, which is less than the symbol rate at 20 kHz. This aligns with (27) that the symbol rate is not related to the decoding.
**Algorithm 2.** DeChirp**Inputs:**  n-bit multi-slope chirp signal, symbol_rate, VCO’s_gain **Output:** Recovered information in APAM formatFind the first-, second- and third-time derivative of the input signal $y_{0},$ yielding in $y_{1}$, $y_{2}$ and $y_{3}$ respectivelyPerform $\left(y_{1}y_{2}-y_{0}y_{3}\right)\frac{\sqrt{y_{1}^{2}-y_{0}y_{2}}}{y_{1}^{2}-3y_{0}y_{2}}$ get $y_{o}$Scale $y_{o}$ to the factor of ${\left(\left(2^{n}-1\right)\pi k_{f}R_{s}\right)}^{-1}$ and then rounding, resulted in the recovered information in APAM format

### 4.3. SAGC-Based Decoding Simulations

Algorithm 3 is used to implement the demodulation of the multi-slope chirp signal over the AWGN channel.
**Algorithm 3.** SAGC-based multi-slope chirp signal decoding**Inputs:** multi-slope chirp signal, bit_per_symbol **Output:** Recovered information in APAM formatApply an analog Butterworth BPF order 8 at 1 MHz center frequency with 60 kHz span to the input, get the output in time-indexed row vector **Y1**Apply a comparator at 0 V to **Y1** and saturate it by limiter to −1 V and 1 V, get **YL**Apply an analog Butterworth BPF order 8 at 1 MHz center frequency with 600 kHz span to **YL**, get the output **Y2**Take **Y2** to SAGC-2 demodulator, retrieve **Y_pink**Apply analog Bessel LPF order 15 at 33 kHz cutoff frequency to **Y_pink**, get **Y_green**Differentiate **Y_green**, resulted in **Y_blue**Start sample **Y_blue** from peak of the first pilot then, stepping to the next symbolGiven bit_per_symbol as a variable nCalculate average gains need for all 16 pilots, it has constant values at $\pm \left(2^{n}-1\right)$Map other samples according to the average gain, get the final product as a recovered information in APAM format

**Figure 14:**
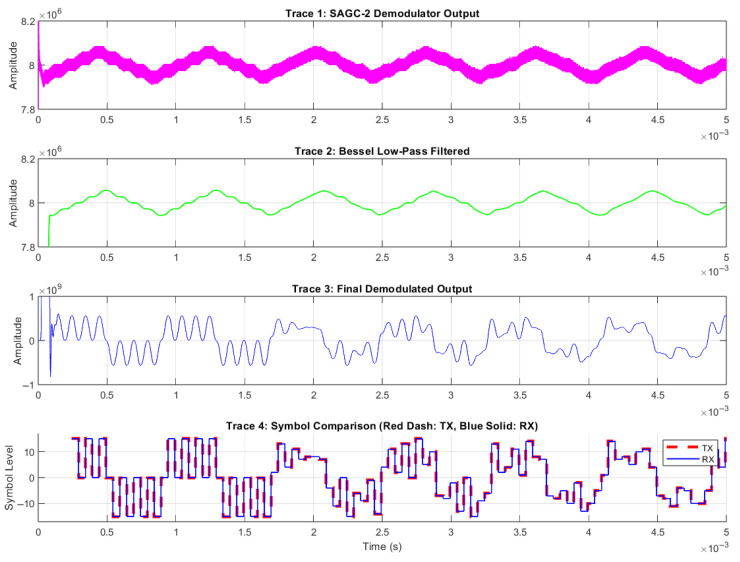
Multi-slope chirp signal demodulation.

When the multi-slope signal had been received, an RF filter and a limiter remove the channel noise from the incoming chirp signal. Next, SAGC-2 functions as a demodulator to retrieve the chirp symbol, as illustrated in the first trace of Figure 14. Next, the Bessel LPF cleans the symbol, resulting in the second trace. By employing the derivative, the slope of the cleaned chirp symbol is detected, as shown in the third trace. Finally, the mapping procedure recovers the APAM signal compared to the transmitted one, as depicted in the last trace.

The optimum cutoff frequency of the Bessel filter is based on the harmonics of SAGC-2; in order to preserve the shape of the signal, several harmonics are kept. The calculations are based on a geometric mean between the symbol rate and the average bandwidth of 2-, 3-, and 4-bit multi-slope chirp signals, with an additional 10% manual adjustment, as shown in Equation (48), found to be the best fit to cover all three signals.

(48) $$f_{c,opt}=1.1\sqrt{R_{s}\times BW}$$

The average bandwidth of 2-, 3-, and 4-bit multi-slope chirp signals is obtained as 45.17 kHz; by substituting for it, the optimum cutoff frequency will be 33 kHz.

### 4.4. Self-Error Correction Results

The errors used in this result have come from the SAGC-based decoding simulation of the 4-bit multi-slope chirp signal over the AWGN channel using Algorithm 4. To maintain the stability of the algorithm, only the pairs that have the offset of 1 and 2 were evaluated. Table 2 shows the logic of the correction.
**Algorithm 4.** Self-error correction**Inputs:** Recovered information in APAM format, bit_per_symbol, error_distance **Output:** Recovered information in APAM format with correctionsGiven bit_per_symbol as a variable nLet **MAX** equal to $2^{n}-1$Take every 2 consecutive symbols as a pair **P**Sum the symbols value inside **P** get **S**Evaluate the offset $E=\left|S-MAX\right|$, if E=1, the symbol with higher error_distance in **P** must be changed its value to satisfy S=MAX, but if E=2 both symbols in **P** are shifted by 1 to satisfy S=MAX, others value of **E** will not be consideredTrigger on every next 2n index until the end of the input, MAX is multiplied by −1

### 4.5. Bandwidth Measurements

**Figure 15:**
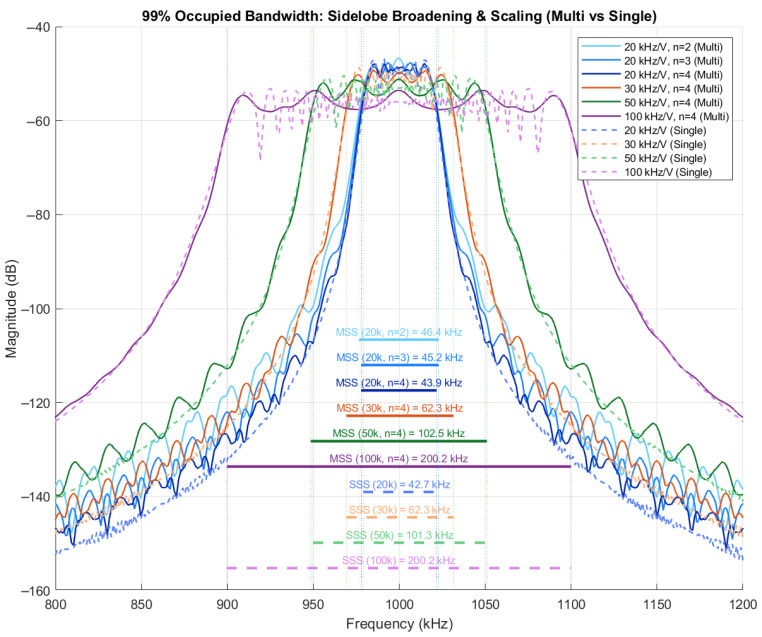
Magnitude spectrum of multi-slope chirp signal.

Four 4-bit multi-slope chirp signals are constructed using Algorithm 1, and all of them have the same center frequency at 1 MHz from the same chirp symbol amplitude of 2 Vpp, with no offset. Their VCO gains ($k_{f})$, however, are different, namely, 20, 30, 50, and 100 kHz/V, labeled in blue, orange, yellow, and purple trace, respectively. Fast-Fourier transform (FFT) resolution of 1.22 kHz is applied to all signals, its magnitude spectrum is plotted, and its bandwidth is measured using the 99% occupied bandwidth method, as shown in Figure 15. The measured bandwidth is summarized in Table 3. It agrees well with (17) in that the bandwidth is twice the VCO gains, but there is a slight shift from the measurement and a sharp transition between the symbols of the multi-slope chirp signal observed in lower-bit-per-symbol signals, which causes a stronger transition than the higher-bit-per-symbol signals.

The sidelobes of the multi-slope signal appear because it contains multiple slopes in one cycle while the single-slope signal has only one slope, so its high-frequency sinusoidal components are more than those of the single-slope signal; it is like a continuous ramp signal and staircase ramp signal, and finally it becomes sidelobe harmonics, as shown in Figure 15. However, its effective bandwidth has a small change.

### 4.6. Error Performance

**Figure 16:**
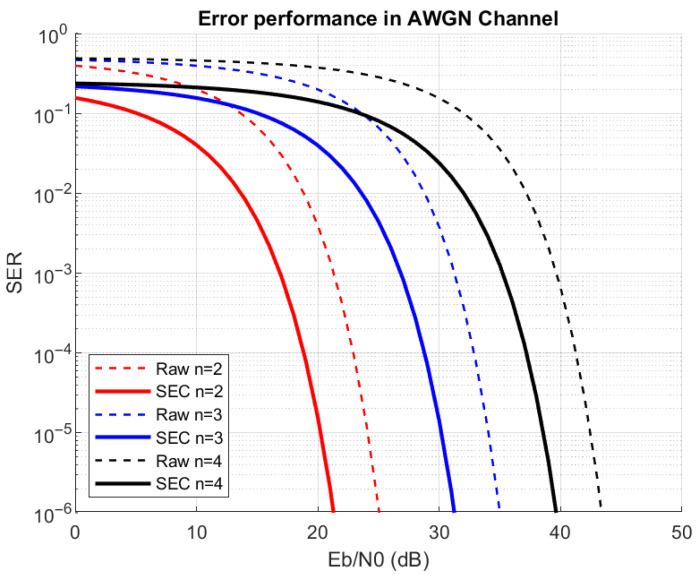
Error performance of proposed receiver of multi-slope chirp signal over AWGN channel before SEC (solid) and after SEC is made (dashed).

The 2-, 3-, and 4-bit multi-slope chirp signals with a center frequency of 1 MHz, a VCO gain of 20 kHz/V, a symbol rate of 20 kHz from the chirp symbol amplitude of 2 Vpp, and with no offset are generated to test performance over the AWGN channel. The results shown in solid trace mean that self-error correction is applied while those in dash trace indicate that it is not. Performance is calculated using the symbol error rate (SER) and is plotted in Figure 16. From (41), the normalized minimum Euclidean distances for the 2-, 3-, and 4-bit multi-slope chirp signals are 0.2657, 0.0846, and 0.0322, respectively.

**Figure 17:**
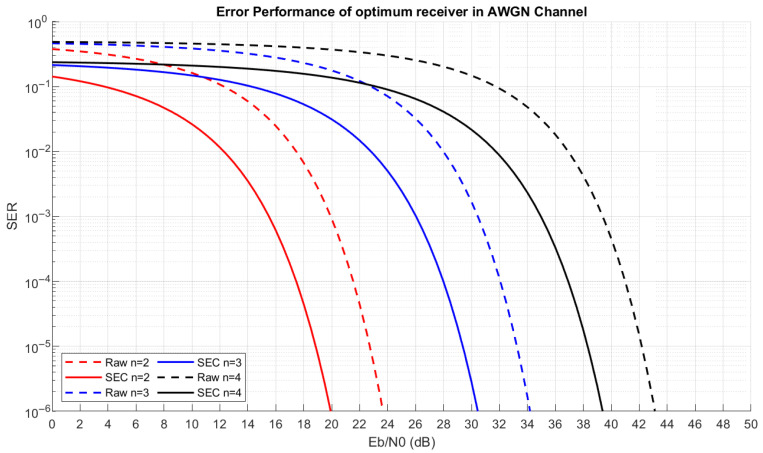
Error performance of optimum receiver of multi-slope chirp signal over AWGN before SEC (solid) and after SEC is made (dashed).

For the optimum receiver, the normalized minimum Euclidean distance is found to be 0.44, 0.131, and 0.047 for the 2-, 3-, and 4-bit multi-slope chirp signals, respectively. Thus, the optimum error performance can be plotted in Figure 17.

## 5. Discussion

In this study, multi-slope chirp modulation is proposed as an alternative modulation scheme for continuous-communication LPWANs, offering faster Time-on-Air and more bandwidth efficiency in a trade-off with higher SNR requirements for a system that does not need to communicate over a very long distance, but a faster data rate and efficiencies are required. The transmitter works quite straightforwardly by having APAM and then integrating it into the symbol before sending it to the VCO. Demodulation is presented for both lossless and AWGN channels. The DeChirp algorithm is introduced to blindly detect the information in lossless channels. However, in the noisy channel, SAGC-2 is used to clean the noise and demodulate the signal back to the symbol.

The bandwidth of the multi-slope chirp signal can be easily controlled by tuning VCO’s gain or adjusting the amplitude of the chirp symbol; the bandwidth can remain consistent due to the Manchester encoding but it has to trade with 2× overhead. Researchers have suggested that more efficient line coding that provides DC balance, such as 3b/4b or 8b/10b line coding [22], could also be used but the bipolar clock has to be adjusted to cover the period that DC will be balanced before the integration; this will improve the spectral efficiency of the signal.

**Figure 18:**
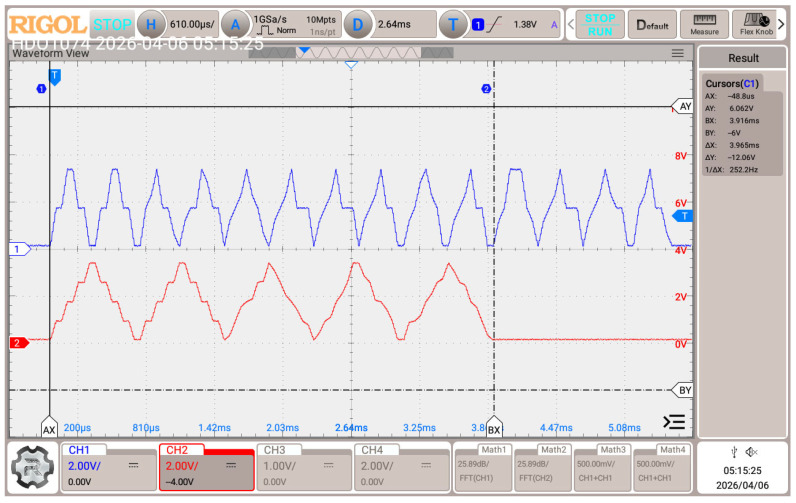
Time-on-Air comparison between 2-bit (blue) and 4-bit (red) multi-slope chirp symbols.

Next, 2-bit and 4-bit multi-slope chirp symbols are generated using the NUCLEO-G474RE development board, which includes two built-in DACs, to evaluate the hardware implementation capabilities and to measure the Time-on-Air. The payload is a 12-byte string “-HelloWorld-”. The output signal is captured by an oscilloscope, as depicted in Figure 18. The measured ToA includes two pilot cycles, which is the preamble for the mapping process, aligning with the result shown in Figure 18. The measured ToA results agree well with the calculations in Table 4.

A comparison of the results in Table 4 and Table 5 and Figure 18 shows that the ToA of MSS is shorter compared to LoRa; by switching from LoRa SF 6 to 2-bit MSS, the communication will have ~11× more bandwidth efficiency, along with a reduction in the Time-on-Air by half, but with more SNR required. Moreover, a higher order of MSS can be used to achieve more spectral efficiency, as shown in Table 5, but it requires more SNR, as shown in Figure 16 and Figure 17. Additionally, SEC can be applied to achieve better error performance, but the author suggests replacing Algorithm 4 with the soft-decision Viterbi decoder instead of hard-coded error distance to optimize the SEC and improve error performance.

Although all of the presented symbol error rate (SER) curves are analytically supported by mathematical models, an empirical RF hardware validation of this study is still required to verify these SER curves in a real-world environment. Conventionally, the receiver’s optimum performance is derived using a matched filter, whereas the performance of the proposed receiver is derived from noise variance generated by the system’s differentiator. Future research will turn the simulations into physical hardware implementations using software-defined radio (SDR) units, to evaluate performance against real-world multipath interference, especially for the absolute SNR needed to achieve an acceptable SER.

## Figures and Tables

**Table 1:** Linear parameters of n-bit multi-slope chirp symbols (n-MSC) in the first half cycle.

n-MSC Equation	Data Symbols	Redundant Symbols
$m_{i,n}\left(t\right)=k_{ci,n}t+v_{i,n}$	$\left(j-1\right)T_{s}\leq t<\left(j-0.5\right)T_{s}$	$\left(j-0.5\right)T_{s}<t\leq jT_{s}$
Slope $\left(k_{ci,n}\right)$	$\frac{4d_{i}}{n\left(2^{n}-1\right)T_{s}}$	$\frac{4\left(\left(2^{n}-1\right)-d_{i}\right)}{n\left(2^{n}-1\right)T_{s}}$
y-interception $\left(v_{i,n}\right)$	$\frac{2\left(j-1\right)\;[\left(2^{n}-1\right)-2d_{i}]}{n\left(2^{n}-1\right)}-1$	$\frac{2j\left[2d_{i}-\left(2^{n}-1\right)\right]}{n\left(2^{n}-1\right)}-1$

**Table 2:** Error correction logic.

Symbol No.	Decoded Symbol	Error Distance	Corrected Symbol	Transmitted Symbol
1	−2	0.4466	−1	−1
2	−14	0.0124	X ^1^	−14
3	15	0.4045	14	14
4	1	0.2624	X ^1^	1
5	7	X ^2^	6	6
6	10	X ^2^	9	9

^1^ X = No correction was performed due to lower error distance in the pair. ^2^ X = It does not matter due to E=2.

**Table 3:** Bandwidth comparison between multi-slope and single-slope chirp signals.

Bits per Symbol	$k_{f}$ (kHz/V)	Multi-Slope Chirp Signal Bandwidth (kHz)	Single-Slope Chirp Signal Bandwidth (kHz)	Offset
2	20	46.39	42.72	8.59%
3		45.17		5.73%
4		43.95		2.88%
4	30	64.70	62.26	3.91%
4	50	104.98	101.32	3.61%
4	100	203.86	200.2	1.83%

**Table 4:** Transmission time calculations; the symbol period is 50 μs.

#Data Byte	#Encoded Binary	Bits per Symbol	#PAM Symbols	#Data Cycle	#Pilot Cycle	#Total Cycle	#Total Symbols	ToA
12	192	2	96	12	2	14	112	5.6 ms
		3	64	5.33	2	7.33	88	4.4 ms
		4	48	3	2	5	80	4 ms

**Table 5:** Time-on-Air and effective spectral efficiency ($\eta_{e}$) comparison between multi-slope chirp signal and LoRa, where Time-on-Air is measured over 50 bytes of payload with its overhead.

Article	Modulation	Coding Rate	$\eta_{e}$ (bps/Hz)	ToA (ms)
Proposed	4-bit MSS ^1^	1/2 ^2^	3.644	20
	3-bit MSS		1.770	22
	2-bit MSS		0.862	28
[23,24]	LoRa SF = 6	4/5	0.075	57.47
	LoRa SF = 12		0.002	2203.60

^1^ MSS = multi-slope chirp signal with self-error correction; ^2^ Manchester coding rate.

## Data Availability

The data supporting the findings of this study can be requested from the corresponding author.
